# Supplementary material for: A Critical View of the Application of the APEX Software (Aqueous Photochemistry of Environmentally-Occurring Xenobiotics) to Predict Photoreaction Kinetics in Surface Freshwaters
Source: Molecules. 2019 Dec 18;25(1):9. doi: 10.3390/molecules25010009 (PMC7017383; doi:10.3390/molecules25010009)
Supplement: Supplementary file 1 [file molecules-25-00009-s001.zip › 3.2.4_gcc-4.4.0.zip!/3.2.4_gcc-4.4.0/3.2.4_gcc-4.4.0/doc/octave/html/interpreter/Function-Locking.html]

Function Locking - Untitled


Next: Function Precedence,
Previous: Overloading and Autoloading,
Up: Function Files


---

#### 11.7.5 Function Locking

It is sometime desirable to lock a function into memory with the
`mlock` function. This is typically used for dynamically linked
functions in Oct-files or mex-files that contain some initialization,
and it is desirable that calling `clear` does not remove this
initialization.

As an example,

```
     mlock ("my_function");
```

prevents `my_function` from being removed from memory, even if
`clear` is called. It is possible to determine if a function is
locked into memory with the `mislocked`, and to unlock a function
with `munlock`, which the following illustrates.

```
     mlock ("my_function");
     mislocked ("my_function")
     => ans = 1
     munlock ("my_function");
     mislocked ("my_function")
     => ans = 0
```

A common use of `mlock` is to prevent persistent variables from
being removed from memory, as the following example shows:

```
     function count_calls()
       persistent calls = 0;
       printf ("'count_calls' has been called %d times\n",
               ++calls);
     endfunction
     mlock ("count_calls");
     
     count_calls ();
     -| 'count_calls' has been called 1 times
     
     clear count_calls
     count_calls ();
     -| 'count_calls' has been called 2 times
```

It is, however, often inconvenient to lock a function from the prompt,
so it is also possible to lock a function from within its body. This
is simply done by calling `mlock` from within the function.

```
     function count_calls ()
       mlock ();
       persistent calls = 0;
       printf ("'count_calls' has been called %d times\n",
               ++calls);
     endfunction
```

`mlock` might equally be used to prevent changes to a function from having
effect in Octave, though a similar effect can be had with the
`ignore_function_time_stamp` function.

— Built-in Function: **mlock** ()  
> Lock the current function into memory so that it can't be cleared.
>
>
>
> **See also:** munlock, mislocked, persistent.

— Built-in Function: **munlock** (fcn)  
> Unlock the named function. If no function is named
> then unlock the current function.
>
>
>
> **See also:** mlock, mislocked, persistent.

— Built-in Function: **mislocked** (fcn)  
> Return true if the named function is locked. If no function is named
> then return true if the current function is locked.
>
>
>
> **See also:** mlock, munlock, persistent.
